# Controllable Multimodal Actuation in Fully Printed Ultrathin Micro-Patterned Electrochemical Actuators

**DOI:** 10.1021/acsami.3c19006

**Published:** 2024-01-24

**Authors:** Ji Zhang, Qingshen Jing, Tom Wade, Zhencheng Xu, Liam Ives, Diandian Zhang, Jeremy J. Baumberg, Sohini Kar-Narayan

**Affiliations:** † Department of Materials Science & Metallurgy, University of Cambridge, 27 Charles Babbage Road, Cambridge CB3 0FS, U.K.; ‡ NanoPhotonics Centre, Cavendish Laboratory, University of Cambridge, JJ Thomson Avenue, Cambridge CB3 0HE, U.K.; § James Watt School of Engineering, University of Glasgow, Glasgow G12 8LT, U.K.

**Keywords:** microactuator, electrochemical actuator, aerosol jet printing, patterning, microfabrication, trilayer actuator

## Abstract

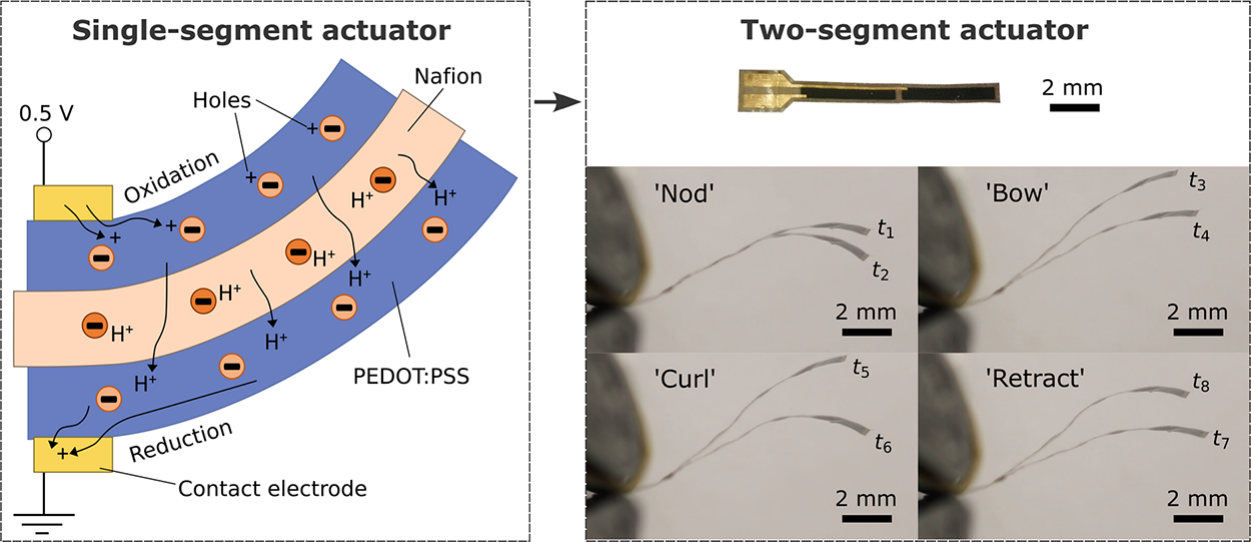

Submillimeter or micrometer scale electrically controlled soft actuators have immense potential in microrobotics, haptics, and biomedical applications. However, the fabrication of miniaturized and micropatterned open-air soft actuators has remained challenging. In this study, we demonstrate the microfabrication of trilayer electrochemical actuators (ECAs) through aerosol jet printing (AJP), a rapid prototyping method with a 10 μm lateral resolution. We make fully printed 1000 × 5000 × 12 μm^3^ ultrathin ECAs, each of which comprises a Nafion electrolyte layer sandwiched between two poly(3,4-ethylenedioxythiophene) polystyrene sulfonate (PEDOT:PSS) electrode layers. The ECAs actuate due to the electric-field-driven migration of hydrated protons. Due to the thinness that gives rise to a low proton transport length and a low flexural rigidity, the printed ECAs can operate under low voltages (∼0.5 V) and have a relatively fast response (∼seconds). We print all the components of an actuator that consists of two individually controlled submillimeter segments and demonstrate its multimodal actuation. The convenience, versatility, rapidity, and low cost of our microfabrication strategy promise future developments in integrating arrays of intricately patterned individually controlled soft microactuators on compact stretchable electronic circuits.

## Introduction

The pursuit of wearability, biocompatibility, and safety in human-machine interaction has led to the growth of stretchable electronics and soft robotics. Several types of electrically driven soft actuators have been extensively studied, including electrostatic, electrothermal, piezoelectric, and electrochemical actuators.^1−3^ Among these, electrochemical actuators (ECAs), which convert electrical energy to mechanical energy via electrochemical processes, have attracted considerable attention due to their ability to achieve large deformations at low actuation voltages.^4,5^ A bendable ECA that operates in air typically comprises a trilayer structure, consisting of a polymer electrolyte sandwiched between a pair of electrodes (Figure S1A); its actuation is caused by ion redistribution and reversible injection of charges toward the electrodes under an applied electric field.^4^ The electrodes are commonly made of metal (as in ionic polymer–metal composites, IPMCs), conductive polymer, or carbon-based materials. A typical conductive polymer electrode is poly(3,4-ethylenedioxythiophene) doped with poly(styrene sulfonate) (PEDOT:PSS) (Figure S1B), a p-type semiconductor exhibiting mixed conduction of ions and holes.^6^ An example of a well-tested polymer electrolyte is Nafion, which contains sulfonate-terminated perfluoroether side chains attached to polytetrafluoroethylene backbones (Figure S1C) and conducts protons in its hydrated acid form.^7^

Despite the immense interest in microrobots due to their vast potential in biomedical applications,^8^ miniaturization of soft actuators has remained a challenge,^9^ and microscale robotic systems capable of complex motions with multiple individually controlled actuating components are yet to be developed. Pioneering works on microfabriated polypyrrole bilayer ECAs^10−12^ proved the feasibility of such aspirations, but those polypyrrole actuators worked only in electrolytic solutions. Microactuators recently developed by Tyagi et al.^13,14^ were also either exclusively aquatic or moisture-driven. Trilayer ECAs can operate in air thanks to the incorporation of a polymer electrolyte, but their micropatterning is more difficult, requiring patterning and aligning electrodes on both sides of the polymer electrolyte while avoiding short circuits between both neighboring and opposing electrodes.

Trilayer ECAs are usually patterned by masking of the polymer electrolyte membrane prior to electrode deposition or selective electrode removal after deposition.^15^ IPMCs with patterned electroless-plated metal electrodes have been fabricated by masking Nafion membranes with tapes^16^ or photoresists,^17,18^ but the swelling of Nafion in water and the dendritic structure at the electrode–electrolyte interface can limit the achievable feature size.^18^ Selective removal methods such as manual scratching,^19^ machine milling,^19,20^ or laser ablation^19,21^ are also challenging for smaller and thinner ECAs, as they require adequately segmenting the electrodes without cutting through the membrane. Although trilayer ultrathin (<20 μm) microactuators have been fabricated by laser processing^22^ and reactive ion etching,^23,24^ these were cut out completely as single-segment actuators and the electrodes were not patterned. On the other hand, photolithography with physical vapor deposition of metal electrodes allows smaller feature sizes for both single-segment and multisegment trilayer ECAs^25,26^ if the swelling of the polymer electrolyte in developer solutions is addressed. Conductive polymers like polypyrrole can be electropolymerized on photolithographically patterned metal electrodes to create conductive polymer trilayer actuators with individually addressed segments;^27,28^ however, two-segment actuators fabricated with this method performed unsatisfactorily (tip displacement of 100 μm when a 5 × 10 mm^2^ segment was actuated at ±1 V, 0.05 Hz), requiring further optimization and downscaling.^27^ In addition, the time, cost, and wastefulness of repeatedly fabricating new designs impede the development and optimization of lithographically patterned ECAs.

Additive manufacturing methods are alternatives to selective masking and selective removal methods, allowing rapid prototyping with great customizability. Several additive manufacturing methods have been employed to fabricate ECA components, including fused filament fabrication (FFF) of Nafion electrolytes,^29^ direct ink writing (DIW) of ionic liquid (IL)-polyvinylidene fluoride (PVDF) blend,^30^ inkjet printing (IJP) of Nafion microfeatures,^31^ IJP of PEDOT:PSS,^32,33^ and DIW of Fe(Tos)_3_ for subsequent vapor phase polymerization (VPP) of PEDOT.^34^ The resolutions of these methods and dimensions of printed features are listed in Table S1. In these studies of additively manufactured trilayer ECAs, each printing technique was used to fabricate either the electrode or the polymer electrolyte, but not the whole device. The reliance on other fabrication methods in between or after printing reduced the overall prototyping speed and compromised the precision of fabrication of part of the device, especially if the soft polymer membrane required flipping. Therefore, the use of a single additive manufacturing method to print and pattern every layer of the device would be advantageous.

Here, we report, for the first time, an ECA microfabrication technique based entirely on aerosol jet printing (AJP). AJP is an additive manufacturing technique that allows rapid prototyping of different geometries of a large variety of materials with 10 μm lateral resolution^35−39^ and fabrication of freestanding multilayer structures with accurate alignment.^40^ With a simple, low-cost, and rapid sequential deposition procedure, we construct 1000 × 5000 × 12 μm^3^ fully aerosol-jet-printed PEDOT:PSS/Nafion/PEDOT:PSS trilayer actuators that are capable of operating in air at low voltages (around 0.5 V). We also fully print actuators with two individually controlled segments and demonstrate their multimodal actuation.

The novelty in our work is that all parts of the actuator are aerosol-jet-printed with microscale resolution (Table S1), including a Nafion electrolyte, which is aerosol-jet-printed for the first time. A layer-by-layer printing approach enables the fabrication of both ultrathin microactuators and micropatterned actuators with individually addressable segments. Since the ink aerosol dries almost immediately as it contacts the heated substrate, waiting time between printing different layers is not required. As a result, fabrication of the trilayer actuator and the two-segment actuator presented in this work takes only ∼3 and 4 h, respectively, including curing postprinting. Being purely bottom-up, our approach generates almost no waste, minimizing costs in research and prototype development. Our approach enables the patterning of multiple aligned layers with microscale precision similar to lithography-based microelectromechanical system fabrication processes while at the same time surpassing lithographic approaches in terms of prototyping speed and material economy.

While metals, conductive polymers, and carbon-based materials are common electrode materials for trilayer actuators and are all potentially printable, we choose the conductive polymer PEDOT:PSS because of its stretchability, wide availability of dispersion, and ease of printing.

## Results and Discussion

### AJP Trilayer Actuator

**Figure 1:**
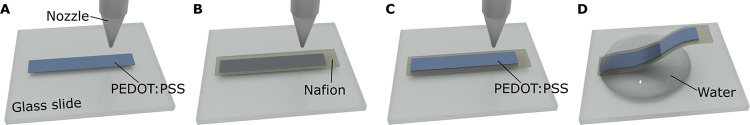
Fabrication procedure of a single-segment PEDOT:PSS/Nafion/PEDOT:PSS trilayer actuator. (A) PEDOT:PSS is printed on a glass substrate heated to 80 °C. (B) Nafion is printed on top of the PEDOT:PSS layer. The substrate is heated to 70 °C, a temperature for drop casting reported in the literature.^41^ Unlike drop casting, Nafion film forms almost instantaneously after printing due to fast evaporation of the solvent in the aerosol, enabling the following steps without waiting. (C) Another PEDOT:PSS layer is printed on top with the substrate heated to 80 °C. The sample is then cured in an oven at 150 °C for 2 h. (D) After curing, the sample is submerged in deionized water and peeled off from the glass substrate using tweezers. The sample is then dried between paper towels.

**Figure 2:**
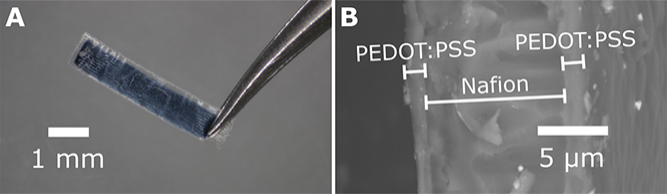
(A) Optical image of a fully printed trilayer actuator held by tweezers. (B) SEM cross-sectional image of the trilayer actuator.

We make fully aerosol-jet-printed PEDOT:PSS/Nafion/PEDOT:PSS trilayer actuators by sequentially printing PEDOT:PSS (bottom layer), Nafion, and PEDOT:PSS (top layer) on a glass slide followed by curing the samples at 150 °C for 2 h (Figure 1, more details in the Experimental Section). The actuator can be peeled off in water because the Nafion film absorbs water readily and expands, losing attachment to the glass slide along with the PEDOT:PSS layer underneath. The thickness and morphology of each layer can be tuned by varying the number of printing passes (loops), line spacing in the fill pattern, aerosol flow rate, and the speed of printing. As shown in Figure S2, in the same batch of samples, the AJP Nafion film thickness is linearly proportional to the number of printing loops with other printing parameters kept constant. However, there may be slight variations between different batches because the amount of aerosol coming out of the nozzle may be affected by numerous other factors such as temperature, amount of ink in the vial, and length and cleanliness of the tube through which the aerosol is transported. This inconsistency can be eliminated by further optimizing and standardizing the printing procedure. In this study, we use five printing loops to print the Nafion layer and two printing loops to print the PEDOT:PSS layer. The resulting actuators (Figure 2A) are 1 × 5 mm^2^ rectangular cantilevers, with a PEDOT:PSS electrode area of 0.7 × 4.35 mm^2^. The trilayer structure can be seen from the scanning electron microscopy (SEM) cross-sectional image (Figure 2B). The thicknesses of the Nafion and PEDOT:PSS layers of the actuators for actuation testing are measured by profilometry and are shown in Table 1 (Sample I has slightly different thickness measurements because it is printed in a different batch). From nanoindentation tests on printed samples of 7.8 μm-thick Nafion and 7.2 μm-thick PEDOT:PSS, the Young’s moduli are *E*_Nafion_ = 2.5 ± 0.5 GPa and *E*_PEDOT:PSS_ = 7.0 ± 0.7 GPa. The errors are due to the surface roughness of the printed samples (RMS roughness of ∼0.8 μm for Nafion and ∼0.2 μm for PEDOT:PSS, calculated from profilometry data). The electronic conductivity of printed PEDOT:PSS is about 0.5 S cm^–1^, as determined by measuring the conductance of printed PEDOT:PSS samples of three different thicknesses on the Nafion substrate (Figure S3). The conductivity and stretchability of pristine PEDOT:PSS are low, but can be drastically improved with additives, which will be explored in future investigations.

**Table 1:** Thicknesses of the Nafion and PEDOT:PSS Layers of the Tested Actuators

actuator name	Nafion layer thickness (μm)	PEDOT:PSS layer thickness (μm)	total thickness (μm)
Sample I	8.6	1.8	12.2
Sample II	7.9	2.1	12.1
Sample III	7.9	2.1	12.1

**Figure 3:**
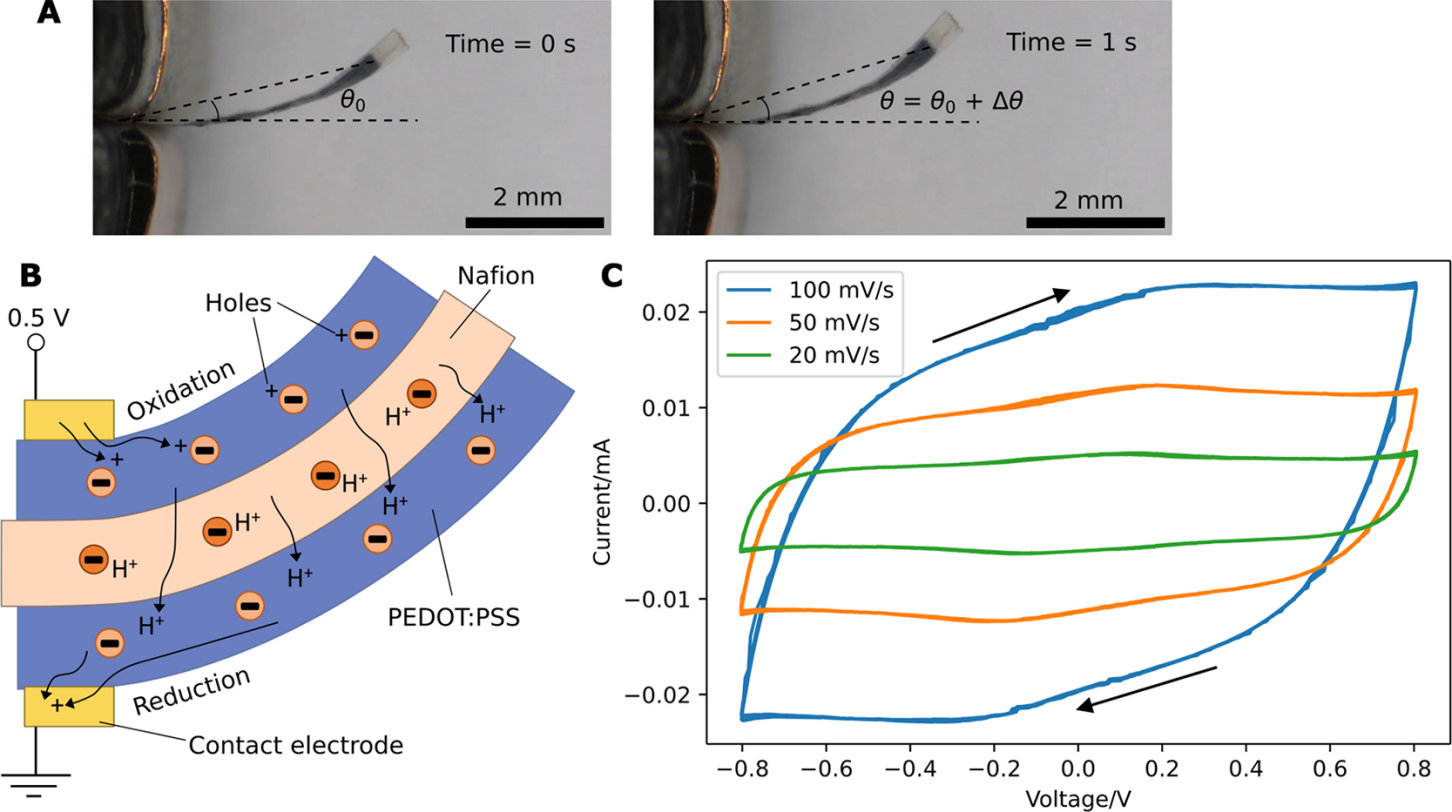
(A) Video frames showing bending angle (θ) of actuator (Sample I) 0 and 1 s after application of 0.5 V. Actuator has initial bending angle of θ_0_. The offset-subtracted angular deflection (Δθ) is calculated by subtracting θ_0_ from θ. In 1 s, Δθ = 0.057 rad (θ_0_ = 0.215 rad, θ = 0.272 rad). (B) Schematic of actuation mechanism in PEDOT:PSS/Nafion/PEDOT:PSS trilayer actuator. (C) CV of actuator (Sample II) measured between ±0.8 V at different scan rates.

The actuators are clamped on one end for actuation testing in air, and the bending angles are obtained through motion tracking of the recorded videos (Figure 3A, more details in the Experimental Section). The actuators usually have an initial bending angle θ_0_, likely because of the slight differences in printing the top and bottom layers (the bottom PEDOT:PSS layer is printed on glass, and the top PEDOT:PSS layer is printed on Nafion) and the slight inconsistencies in controlling the printing parameters. Flatter actuators may be produced by further optimization of the printing conditions. In Figure 3A, 0.5 V is applied to an actuator (Sample I), resulting in extra deflection Δθ and a bending angle θ = θ_0_ + Δθ. The charge transfer and actuation mechanism of the actuator is illustrated in Figure 3B. The sulfonate anions are fixed on the Nafion and PSS polymer chains; the protons (H^+^) are mobile, along with their hydration water molecules. Upon applying voltage, protons migrate from the anode to the cathode, resulting in anode contraction and cathode expansion; hence, the cantilever bends toward the anode. Proton transport in Nafion occurs through both Grotthuss and vehicle mechanisms, commonly explained in a cluster model of Nafion.^42^ At the cathode, proton insertion into PEDOT:PSS is accompanied by reduction and hole extraction from PEDOT: the proton replaces a hole in PEDOT to pair with a sulfonate anion.^43,44^ The opposite process (proton extraction, oxidation, and hole insertion) happens at the anode. The behavior of similar actuators has been modeled and explained in previous papers.^45,46^ The cyclic voltammograms (CVs) of the actuator (Sample II) between ±0.8 V (Figure 3C) are close to parallelogram-shaped with broad redox peaks due to fast faradaic processes, showing pseudocapacitive behavior of the electrodes. By integrating the current–time curves using the trapezoidal rule (Figure S4), the peak-to-peak transported charge is found to be 0.140 ± 0.002, 0.156 ± 0.003, and 0.169 ± 0.005 mC for scan rates of 100, 50, and 20 mV s^–1^, respectively.

**Figure 4:**
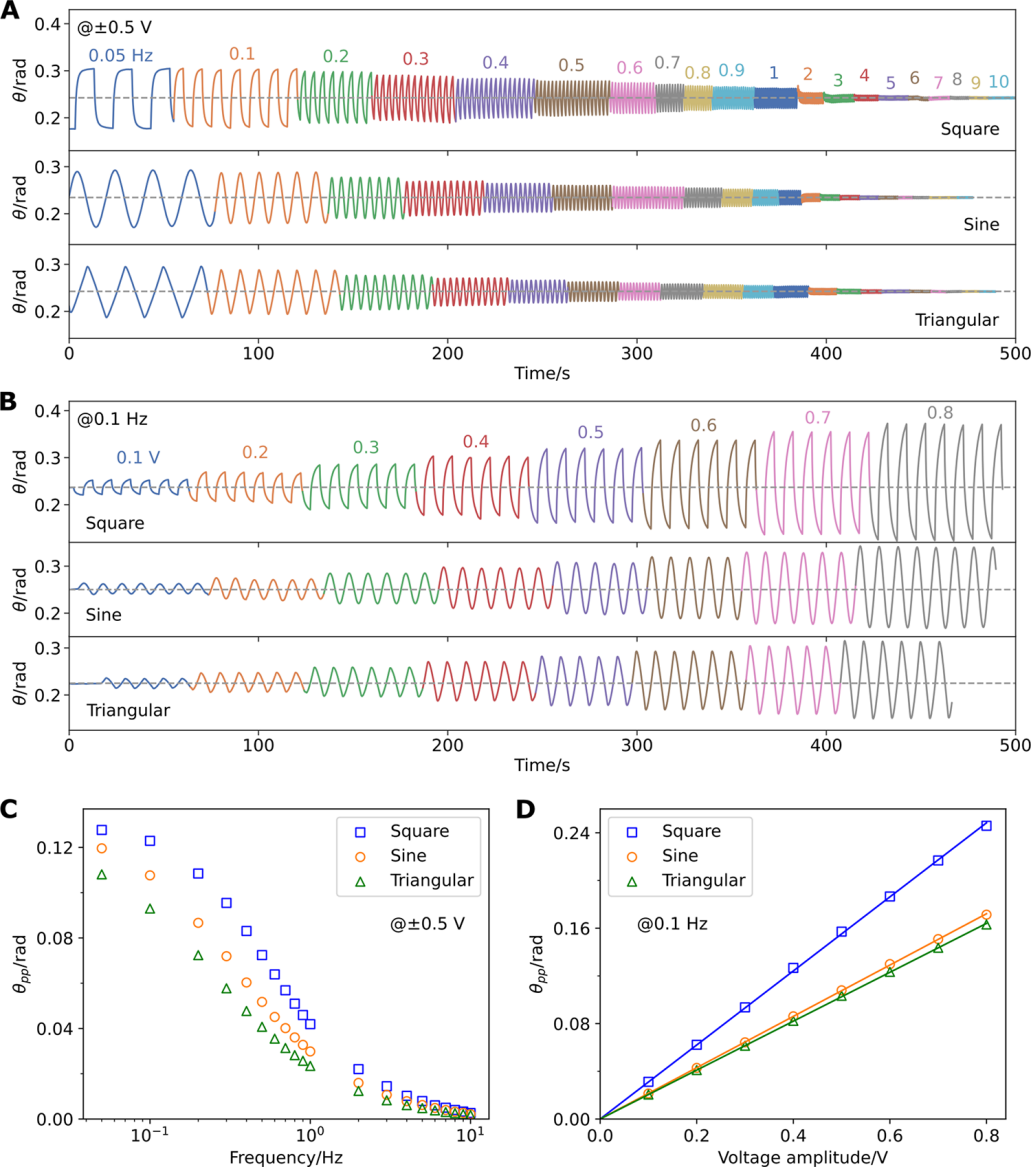
Frequency and voltage sweep tests in air on an AJP trilayer actuator (Sample I). (A) Bending angle (θ) for frequency sweep from 0.05 to 10 Hz with different waveforms of 0.5 V amplitude. (B) θ under voltage sweep from 0.1 to 0.8 V amplitude with different waveforms at 0.1 Hz. (C) Plot of peak-to-peak angular deflection (θ_pp_) against the frequency of applied voltage with an amplitude of 0.5 V. (D) Plot of θ_pp_ against voltage amplitude at a frequency of 0.1 Hz.

We record the actuation of an AJP actuator (Sample I) under ±0.5 V square, sinusoidal, and triangular waves for frequencies ranging from 0.05 to 10 Hz (Figure 4A and Video S1) and under 0.1 Hz square, sinusoidal, and triangular waves of amplitudes ranging from 0.1 to 0.8 V (Figure 4B and Video S2). We determine the transferred charge over time by integrating the current–time graphs using the trapezoidal rule (Figures S5 and S6); for the frequency sweep charge–time graphs, data at higher frequencies are discarded due to limited temporal resolution of current measurement by the digital multimeter. With a voltage amplitude of 0.5 V, the peak-to-peak angular deflection (*θ*_pp_) decreases at higher frequencies (Figure 4C). This is due to the shorter time available for charge transfer to drive the oscillation, as is evident from the decreasing trend of peak-to-peak charge transfer (*Q*_pp_) at higher frequencies (Figure S7A). Deflections can be detected for frequencies up to 10 Hz, after which the vibrations become hardly visible. At a frequency of 0.1 Hz, *θ*_pp_ is found to be linearly proportional to the voltage amplitude, reaching 0.25 rad (14°) at 0.8 V (Figure 4D). *Q*_pp_ follows a similar linear trend (Figure S7B). This linear proportionality has been observed in previous work on trilayer conductive polymer ECAs^47−51^ and agrees with the low voltage region of modeling from a rigid finite element model.^45^ It can be explained by the volumetric capacitive behavior of the PEDOT:PSS electrodes and the linear relationship between electrode volume changes and the exchanged charge at equilibrium,^52,53^ which can be seen from plotting the peak-to-peak deflection against the peak-to-peak charge transfer for the voltage sweep tests (Figure S7D). The relationship is less linear for the frequency sweep tests (Figure S7C) because of the dependence of the mechanical response on the driving frequency. We notice that ambient conditions such as humidity and temperature can considerably affect the actuation performance; hence, actuation results may vary if these parameters are not controlled. However, such effects are small within the same set of data, so the linear trend in Figure 4D can be clearly seen. To demonstrate the durability of the AJP actuator, we actuate a fresh actuator (Sample III) under 0.1 Hz, ±0.5 V square wave for 50 min and notice little drop in performance (Figure S8).

**Figure 5:**
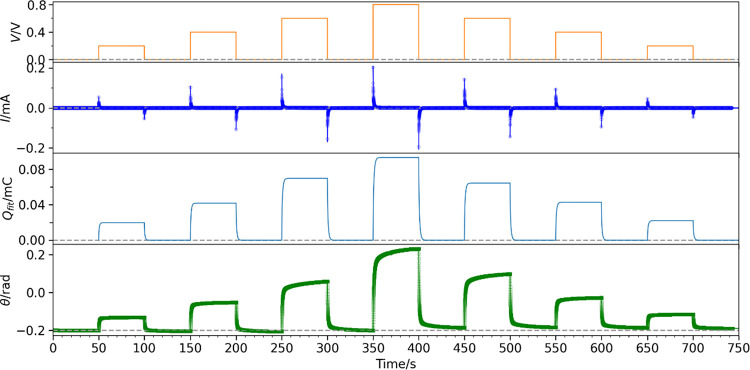
Chronoamperometry response curves of the actuator. *V* is the voltage applied over time. When *V* = 0 V, the actuator is connected to a short circuit. *I* is the current flowing through the actuator. Data points of *I* in each charge–discharge cycle are fitted to two-term exponentials. *Q*_fit_ is the charge stored by the actuator, obtained by integrating the fitted *I* functions with respect to time. θ is the bending angle. Data points of θ in each charge–discharge cycle are also fitted to two-term exponentials. The creep in θ is barely noticeable; the actuator returns to almost the same position after short-circuiting every time.

To characterize the response of AJP actuators under DC, we conduct chronoamperometry^46^ on a fresh actuator (Sample II). The actuator undergoes 50 s of charging under voltages from 0.2 to 0.8 V in steps of 0.2 V followed by 50 s of discharging (connection to a short circuit) (Figure 5). There are seven cycles in total, for which the charging voltage increases and then decreases. Data points of current *I* in each charge–discharge cycle are fitted to two-term exponentials with time constants *t*_1_ and *t*_2_; data points of bending angle θ in each charge–discharge cycle are fitted to two-term exponentials with time constants *t*_3_ and *t*_4_ (see Supporting Information for details). The charge stored by the actuator, *Q*_fit_, is obtained by integrating the fitted current functions. The bending angle–time curve has a shape similar to that of the charge–time curve but appears to respond more slowly to the voltage steps. From the fitted parameters (Figure S9A,D), the time constants do not vary much from 0.2 to 0.8 V; the average time constants that fit the current (*t*_1_ = 0.2 s, *t*_2_ = 1 s) are smaller than the average time constants the fit the deflection (*t*_3_ = 0.5 s, *t*_4_ = 12 s), suggesting a mechanical lag between charge transfer and actuation. Due to the capacitive behavior of the PEDOT:PSS electrodes, the maximum charge stored appears linearly proportional to the applied charging voltage (Figure S9C), and the deflection resulting from charge transfer is also related to the charging voltage with an approximately linear relationship (Figure S9F). Although the curve fitting with two exponentials is empirical and cannot describe the complex actuation behavior exactly, it shows that the transient response is on the order of seconds and that the charge stored and equilibrium deflection are proportional to the applied voltage up to 0.8 V.

Compared to actuators of similar compositions in the literature, our AJP PEDOT:PSS/Nafion/PEDOT:PSS actuators have a low thickness (∼12 μm) and, therefore, relatively fast response and large deflection under low voltages. These features are also evident in ultrathin actuators fabricated with other methods in the literature.^22,23^ A smaller thickness means a larger electric field generated across the actuator at low voltages, shorter lengths for proton transport, and smaller area moments of inertia and flexural rigidity, leading to larger bending curvatures and faster bending speeds of the cantilever when subjected to forces caused by proton migration. For fair comparison with studies that report linear tip deflections instead of angular ones, we calculate their normalized deflection against the cantilever length, which can be assumed to be approximately equal to the angular deflection in radians for small angles. A PEDOT:PSS/Nafion/PEDOT:PSS actuator fabricated with solution-based polymerization had a maximum *θ*_pp_ of ∼0.1 rad under 3 V amplitude AC input.^54^ A PEDOT/Nafion/PEDOT actuator fabricated by polymerization on a surface-treated Nafion membrane achieved ∼0.32 rad deflection under 1.5 V step input but required more than 80 s to get close to equilibrium.^55^ In more recent papers, the PEDOT:PSS/Nafion/PEDOT:PSS configuration was often reported alongside higher-performance ECAs as a benchmark for comparison.^50,51,56−58^ From the data provided by Yu et al.,^57^ a PEDOT:PSS/Nafion/PEDOT:PSS actuator made by drop casting had an equilibrium time >10 s, a θ_pp_ of ∼0.1 rad under 0.1 Hz, ±0.5 V square wave input, and no actuation performance at frequencies above 2 Hz.

### Two-Segment Actuator

**Figure 6:**
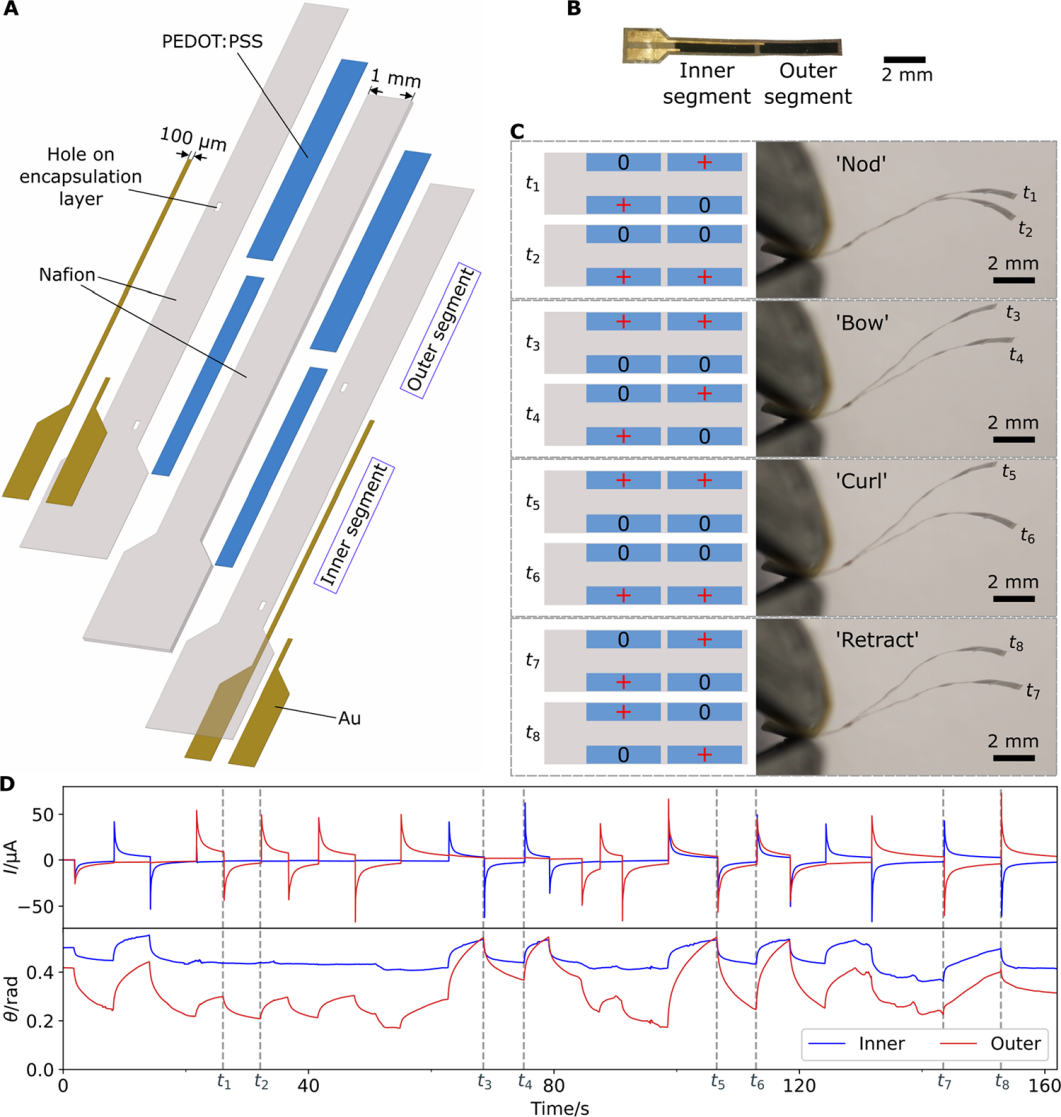
(A) Exploded diagram of a two-segment actuator (thicknesses of all layers are exaggerated 10 times). The thin Au lines have a width of 100 μm. The small rectangular holes in the Nafion encapsulation layer allow connection between the Au lines and corresponding PEDOT:PSS electrodes. (B) Optical image of the two-segment actuator. (C) Multimodal actuation of the two-segment actuator when different voltages are applied to the two pairs of individually addressed electrodes. Schematics on the left show the voltage applied on each electrode at times from *t*_1_ to *t*_8_ (after voltages have been applied for a few seconds). “0” represents 0 V and “+” represents 0.8 V applied on the electrode. Images on the right are overlaid in pairs, showing the deformation of the actuator at both instants indicated. Actuator has an initial curvature as in Figure 3A. (D) Current (*I*) and tip angular displacement (θ, relative to the point of clamping) measurements of the inner and outer segments subject to inputs of different polarities on each segment. Irregular transients occasionally observed are due to air movement and vibrations caused by actuation and manual switch operation.

Exclusively with AJP, we fabricate two-segment actuators by sequentially printing and patterning Au, Nafion, PEDOT:PSS, Nafion, PEDOT:PSS, Nafion, and Au on a glass substrate. We add two thin Nafion layers to encapsulate the PEDOT:PSS electrodes and print Au outside the encapsulation layer as contact pads and connecting lines (Figure 6A,B). The Au lines reach the PEDOT:PSS electrodes through small holes in the encapsulation layer, forming ohmic Au/PEDOT:PSS contacts.^59^ The longer Au line in the actuator has a resistance of ∼10 Ω from the contact pad to the Au/PEDOT:PSS connection. The Au has a microcrack morphology (Figure S10) which gives rise to its stretchability.^60^ Au weakly adheres to the glass slide, enabling easy peel-off in water after printing and curing. The Nafion encapsulation layer strongly bonds to the Nafion electrolyte layer inside and holds the PEDOT:PSS electrodes in place, obviating any PEDOT:PSS delamination issues.

We use Au instead of PEDOT:PSS as our contact electrode for three reasons. First, pristine PEDOT:PSS used in this study has relatively high resistivity compared to common metallic conductors. Second, from our experience, PEDOT:PSS may sometimes get stuck and left on glass in the peel-off step. With Au contact electrodes, PEDOT:PSS functions solely as an active material and is fully encapsulated. Third, since the connecting line to the outer segment goes past the inner segment, applying an opposite potential may actuate a PEDOT:PSS line, antagonizing the motion of the inner segment. Au contacts do not have this issue, which we checked by fabricating and testing a Au/Nafion/Au actuator which has a “threshold voltage” of ∼2 V. In a voltage sweep test (Figure S11), its deflection increases significantly only >2 V, corresponding to additional electrochemical processes beyond 2 V indicated in the CV curve (Figure S11D) and corroborating results of IPMCs with sputtered Au electrodes.^61^ This actuator also undergoes back relaxation (Figure S11B), an issue common in IPMCs^62^ but absent from the PEDOT:PSS/Nafion/PEDOT:PSS actuator. Since we employ Au only as a contact electrode, these actuation characteristics are irrelevant to our two-segment actuator, whose control voltage of 0.8 V is well below the threshold voltage for a Au/Nafion/Au actuator.

As shown in Figure 6C and Video S3, the inner and outer segments of the actuator can be individually controlled by the voltage applied to the top and bottom electrodes of each segment. When the voltage polarity is kept constant on the inner segment and reversed on the outer segment, the outer segment deflects, but the inner segment stays still (“nod”). When the polarity is kept constant on the outer segment and reversed on the inner segment, the inner segment deflects and the curvature on the outer segment remains approximately the same (“bow”). When the polarity is reversed on both segments in the same way, both segments curve in the same direction, resulting in a large overall deflection (“curl”). When the polarity is reversed on both segments in the opposite way, in the case of “retract”, the inner segment curves upward and the outer segment downward. The relationship between the current flow and the tip angular deflection of each segment is shown in Figure 6D.

We have chosen PEDOT:PSS and Nafion in our study because they are biocompatible, widely investigated, and readily available in dispersions. Nevertheless, recently developed materials were shown to dramatically enhance the ionic conductivity in the actuator and the electronic conductivity and the charge storage capacity of the electrodes, leading to significantly improved actuation.^56,57,63,64^ Microfabrication of these materials and the development of new materials with our AJP approach will potentially create open-air microactuators and multisegment actuators with more impressive performance.

Additionally, the design of the two-segment actuator can be made more compact, enabling further downscaling and incorporation of more individually addressed segments. For example, the width of the Au lines could be reduced from 100 to ∼10 μm with a denser fill pattern and a smaller AJP nozzle size such that more lines and hence more segments can be accommodated.

## Conclusions

We have developed an all-printing strategy for fabricating ECAs and demonstrated the rapid prototyping and micropatterning capabilities. We have established AJP as a powerful tool to fabricate batches of microactuator samples of different designs with minimal amounts of ink material, time, and effort. The convenience of AJP in tuning layer thicknesses, altering layer designs, switching ink composition, and adding extra features is particularly useful for exploring the parameter spaces of actuators, investigating the actuation mechanisms, and optimization of actuating devices. We have created ultrathin (∼12 μm) fully aerosol-jet-printed PEDOT:PSS/Nafion/PEDOT:PSS trilayer open-air ECAs, achieving low actuation voltage (∼0.5 V), relatively fast response (in the order of seconds), and relatively large deflection (θ_pp_ = 0.25 rad under 0.1 Hz, ±0.8 V square wave) compared to previously reported trilayer ECAs of similar compositions. The PEDOT:PSS/Nafion/PEDOT:PSS ECAs respond linearly to the applied voltage and do not have back relaxation issues. With AJP aiding material development, microactuators with an even better performance are anticipated.

We have successfully actuated an AJP micropatterned actuator with two individually controlled submillimeter segments. It utilizes 100 μm-wide AJP Au lines for electrical connection to the outer segments, exploiting the stretchability of AJP Au electrodes and the threshold voltage characteristic of Au-based ECAs. All components in this design are nontoxic and biocompatible, and we believe our success in actuating two-segment actuators can kickstart the development of more sophisticated and intricately patterned actuation systems closely interacting with humans. We look forward to the use of AJP in prototyping microactuating systems in soft microgrippers, microfluidic valves and pumps, haptic displays, and biomedical implants.

## Experimental Section

### Materials

The Nafion ink is prepared by diluting Nafion perfluorinated resin solution (5 wt % in mixture of lower aliphatic alcohols and water, Sigma-Aldrich) in a 1:3 volume ratio with deionized water. The PEDOT:PSS ink is Clevios PH 1000 PEDOT:PSS (1.0–1.3 wt % in water, Heraeus). The Au ink is UTDAu25 TE gold nanoink (25% w/v solution in proprietary organic solvents, UT Dots, Inc.). About 2 mL of each ink is transferred into a dedicated AJP ultrasonic atomizer vial.

### Aerosol Jet Printing

We use an Optomec Aerosol Jet 200 Printer for AJP. In each printing session, the ink is ultrasonically atomized to form an aerosol, which is transported to the deposition head in a continuous flow of nitrogen gas. At the deposition head, another stream of nitrogen gas, called the sheath gas, focuses the aerosol stream onto the substrate. The substrate is mounted on a heated and motorized platen, which moves in a programmable pattern and speed. The printing parameters we use for each ink can be found in Table S2. To form continuous films, the areas are covered by rastering with a serpentine or perimeter fill method. We use AutoCAD to create the designs; drawings are available in Figure S12.

Just before printing each material, the amount of ink in the vial is ∼2 mL. The aerosol delivery tube is cut to around 49 cm. The ambient temperature in the printing room is around 18–20 °C. After all samples are printed, we disassemble and clean the movable parts of the printer (including the tip, the deposition head, the aerosol delivery tube, and the atomizer cap assembly) by sonication in Branson Ultrasonics Industrial Strength Concentrate using Branson Bransonic CPX 3800 Ultrasonic Bath, rinsing with DI water, and blow drying with compressed air.

All samples are cured in a Heratherm OGH 60 oven at 150 °C for 2 h after printing. The trilayer actuator and the two-segment actuator are then soaked in deionized water and peeled off carefully from the glass slide with a pair of tweezers when viewed under a stereomicroscope (KERN OZM 544). We then dry the actuators between paper towels.

### Characterization and Actuation Tests

Optical images and videos of the actuators are taken with the KERN OZM 544 stereomicroscope on which a Canon EOS 2000D DSLR camera is mounted. When recording the actuation of the two-segment actuator, a Sigma 105 mm f/2.8 EX DG macro lens is used in place of the stereomicroscope. SEM images are taken with a TM3030 Plus Tabletop Microscope, with a 15 kV accelerating voltage in backscattered electron mode. The cross-section is obtained by mechanically cutting the sample. Thicknesses of the AJP Nafion and PEDOT:PSS layers are measured with a Veeco Dektak 6M profilometer (stylus radius: 12.5 μm; stylus force: 29.4 μN; scan rate: 25 μm s^–1^; for Sample I) and a DektakXT profilometer (stylus radius: 2 μm; stylus force: 29.4 μN; scan rate: 166.7 μm s^–1^; for Sample II and Sample III). Thickness is obtained by averaging the heights along the profile:
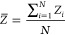


RMS roughness, *Rq*, is calculated using
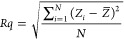


Young’s moduli of Nafion and PEDOT:PSS are measured with an iNano nanoindenter (target load: 25 mN; target depth: 1000 nm; surface approach distance: 2000 nm; surface approach velocity: 100 nm s^−1^; assuming Poisson’s ratio of 0.188). We use the nanoindenter because the small size of the printed samples makes other methods such as extensometer difficult.

The electronic conductivity of PEDOT:PSS on the Nafion substrate is measured with a two-probe multimeter. The probes are connected to two copper strips as contact electrodes, which are pressed onto the deposited PEDOT:PSS, forming a conductor with a length of 3 mm and a width of 0.7 mm. The measurement is repeated for three different thicknesses of PEDOT:PSS. The electronic conductivity is obtained from the gradient of the conductance-thickness curve (Figure S2B).

For electrical and actuation testing, the actuators are clamped with a 3D-printed holder on which copper tape or flat flexible cables (with 1 mm pitch) are affixed for the electrical connection. We try to clamp the actuator as close to the edge as possible every time and, at the same time, avoid short-circuiting between the contact electrodes. When testing for a transient response, voltage is applied with an IT6412 bipolar DC power supply connected to a Devantech USB-RLY08C relay board. The voltage across the actuator is measured by a Keithley 2100 digital multimeter (DMM). Current measurements are obtained by measuring the voltage across a 330 Ω serial resistor with a DMM, or two resistors with two DMMs in the case of a two-segment actuator. The video recording, relay switching, and recording of measurements are all controlled by a LabVIEW (version 21.0.1) program. The testing setup and circuit diagram are shown in Figure S13. Motion tracking is carried out in Blender (version 3.2.0), and the coordinates of the track points are exported with a script on the Blender Python API, enabling bending angle calculations. When the actuation response is tested to voltages of different waveforms and frequencies, an RS Pro RSDG805 function generator is connected to the circuit in place of the DC power supply. CV is carried out in the air by applying triangular waves (±0.8 V for the PEDOT:PSS/Nafion/PEDOT:PSS actuator and ±7 V for the Au/Nafion/Au actuator) and recording the current.

In the actuation of the two-segment actuator, a voltage of 0.8 V is applied by the DC power supply, and the polarity of voltage across each segment is manually controlled by a double pole double throw switch, which makes an H-bridge to switch the current direction (Figure S14).

## Data Availability

Supporting data for this paper is available at the Apollo repository (https://doi.org/10.17863/CAM.105402).

## Supporting Information Available

Curve fitting; list of additive manufacturing methods; printing parameters; chemical structures; plot of thickness against number of printing loops; PEDOT:PSS conductance against thickness; measurements in CV, frequency sweep, voltage sweep, and durability tests; SEM image of AJP Au; Au/Nafion/Au actuator testing results; AutoCAD drawings; circuit diagrams for actuation testing (PDF)
Frequency sweep tests of AJP trilayer actuator (MP4)
Voltage sweep tests of AJP trilayer actuator (MP4)
Actuation of AJP two-segment actuator (MP4)
