# Supplementary material for: Controllable Multimodal Actuation in Fully Printed Ultrathin Micro-Patterned Electrochemical Actuators
Source: ACS Appl Mater Interfaces. 2024 Jan 24;16(5):6485–94. doi: 10.1021/acsami.3c19006 (PMC10859886; doi:10.1021/acsami.3c19006)
Supplement: Supplementary file 1 — am3c19006_si_001.pdf [file am3c19006_si_001.pdf]

Supporting Information

Controllable Multimodal Actuation in Fully Printed Ultrathin Micro-Patterned Electrochemical Actuators

Ji Zhang[1,2], Qingshen Jing[1,3], Tom Wade[1], Zhencheng Xu[1], Liam Ives[1], Diandian Zhang[1], Jeremy J. Baumberg[2], Sohini Kar-Narayan[1,*]

[1] *Department of Materials Science & Metallurgy, University of Cambridge, 27 Charles Babbage Road, Cambridge CB3 0FS, UKs*

[2] *NanoPhotonics Centre, Cavendish Laboratory, University of Cambridge, JJ Thomson Avenue, Cambridge CB3 0HE, UK*

[3] *James Watt School of Engineering, University of Glasgow, Glasgow G12 8LT, UK*

*Email: sk568@cam.ac.uk

Curve Fitting for Chronoamperometry Tests

In the chronoamperometry tests, data points of current I in each charge-discharge cycle are fitted to two-term exponentials with time constants t_1, t_2 and coefficients A_1, A_2:

$$I_{fit} = A_1 e^{-\frac{t}{t_1}} + A_2 e^{-\frac{t}{t_2}} + I_l \qquad \text{(charging)}$$

$$I_{fit} = -A_1 e^{-\frac{t}{t_1}} - A_2 e^{-\frac{t}{t_2}} \qquad \text{(discharging)}$$

The function for charging includes a leakage current term, I_l, to account for the electron transfer across the Nafion membrane, due to the finite electronic conductivity of Nafion and leakage pathways at the clamping point. I_l is about two orders of magnitude smaller than I. By integrating the fitted current functions, the charge stored by the actuator, Q_{fit}, can be obtained:

$$Q_{fit} = A_1 t_1 (1 - e^{-\frac{t}{t_1}}) + A_2 t_2 (1 - e^{-\frac{t}{t_2}}) \qquad \text{(charging)}$$

$$Q_{fit} = A_1 t_1 e^{-\frac{t}{t_1}} + A_2 t_2 e^{-\frac{t}{t_2}} \qquad \text{(discharging)}$$

Similarly, data points of bending angle θ in each charge-discharge cycle are fitted to two-term exponentials with time constants t_3, t_4 and coefficients B_1, B_2:

$$\theta_{fit} = B_1 \left(1 - e^{-\frac{t}{t_3}}\right) + B_2 \left(1 - e^{-\frac{t}{t_4}}\right) + \theta_0 \qquad \text{(charging)}$$

$$\theta_{fit} = B_1 e^{-\frac{t}{t_3}} + B_2 e^{-\frac{t}{t_4}} + \theta_0 \qquad \text{(discharging)}$$

The maximum amount of charge stored under a certain voltage is hence $(A_1 t_1 + A_2 t_2)$, and the maximum deflection is $(B_1 + B_2)$.

Table S1. Additive manufacturing methods for ECA fabrication

Ref.	Method	Printed component	Material	Lateral resolution	Printed feature sizes in ECA
[29]	FFF	Electrolyte	Nafion	1750 μm (extruded filament diameter)	11.5 × 30.5 × 1.0 mm^3 (single segment)
[30]	DIW	Electrolyte	IL-PVDF blend	500 μm (needle diameter)	2 × 12 mm^2 × 60 μm (single segment)
[31]	IJP	Microscale dots on electrolyte	Nafion	~40 μm	43-51 μm dot diameter, 87-92 μm inter-dot spacing, ~2 μm height (single segment)
[33]	IJP	Electrode	PEDOT:PSS	~40 μm	0.8 × 5 mm^2, <10 μm thick (single segment)
[32]	IJP	Electrode	PEDOT:PSS	~40 μm	2 × 20 mm^2 × 2.2 μm (single segment) (multi-segment possible but not demonstrated)
[34]	DIW	Oxidant (for VPP of electrode)	$Fe(Tos)_3$ (for VPP of PEDOT)	~1000 μm	1 × 10 mm^2 (single segment) (multi-segment possible but actuation not demonstrated)
**This work**	AJP	Electrolyte	Nafion	~10 μm	1 × 5 mm^2 × 8.6 μm (single segment), 1 mm wide with 100 × 200 μm^2 holes (2-segment)
		Electrode	PEDOT:PSS	~10 μm	0.7 × 4.35 mm^2 × 1.8 μm (single segment), 0.4 × 4.6 mm^2 with 0.4 mm spacing (2-segment)
		Contact electrode	Au	~10 μm	100 μm wide (2-segment)

Table S2. Printing parameters of the inks.

		**Nafion**	**PEDOT:PSS**	**Au**
Atomiser		Ultrasonic	Ultrasonic	Ultrasonic
Atomiser current		Maximum (~0.6 mA)	Maximum (~0.6 mA)	Maximum (~0.6 mA)
Nozzle size		300 μm	300 μm	300 μm
Platen temperature		70 °C	80 °C	60 °C
Chiller temperature		20 °C	20 °C	20 °C
Sheath flow rate/sccm		160 sccm	140 sccm	140 sccm
Ink flow rate		32 sccm	38 sccm	16 sccm
Printing speed		5 mm/s	3 mm/s	5 mm/s
Raster line separation		20 μm	20 μm	20 μm
No. of loops	PEDOT:PSS/Nafion/ PEDOT:PSS actuator (Samples I, II, and III)	5 loops	2 loops per layer	N.A.
	Au/Nafion/Au actuator	5 loops	N.A.	1 loop per layer
	Two-segment actuator	5 loops as body, 1 loop per encapsulation layer	2 loops per layer	1 loop per layer
	Nafion nanoindentation sample	5 loops	N.A.	N.A.
	PEDOT:PSS nanoindentation sample	N.A.	10 loops	N.A.

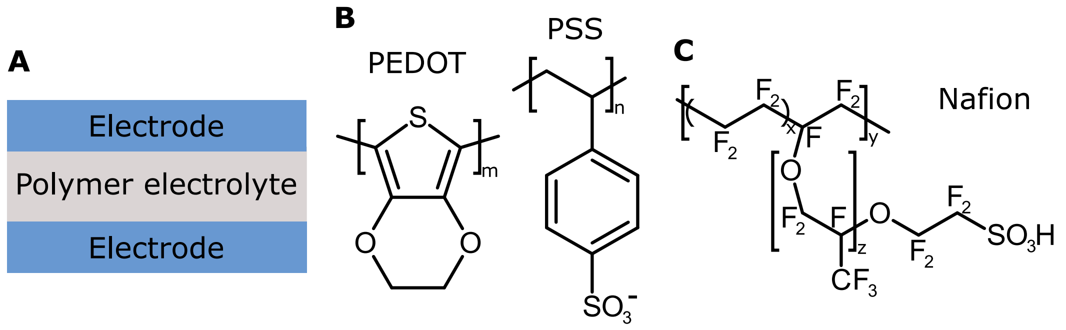

Figure S1. (A) Trilayer structure of ECA. **(B)** Chemical structure of PEDOT:PSS electrode. **(C)** Chemical structure of acid form Nafion electrolyte.

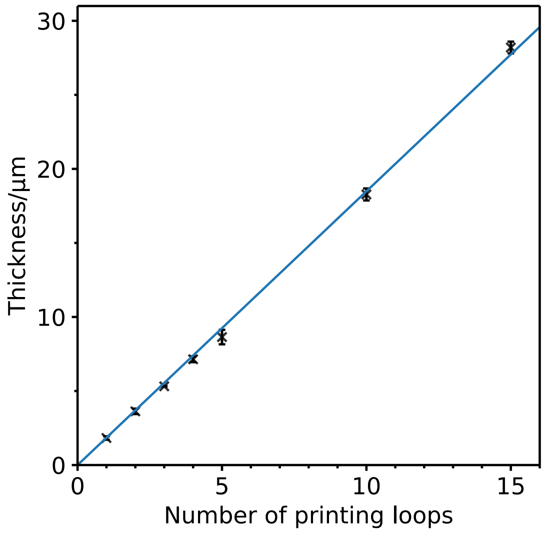

Figure S2. Relationship between AJP Nafion film thickness and number of printing loops. The thickness of each sample is obtained from 3 profilometry measurements. The error bars indicate the sample standard deviation. The trend line is fitted through the origin, showing the linear proportionality.

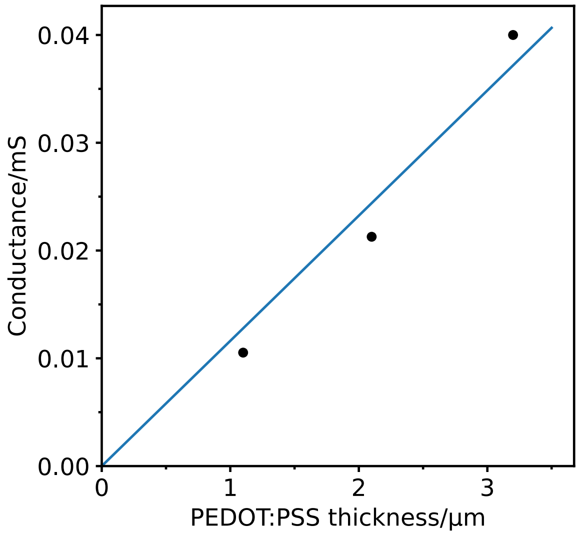

Figure S3. Conductance of PEDOT:PSS of different thicknesses on Nafion substrates. The conductance is measured from two ends of the deposited PEDOT:PSS, forming a conductor with length of 3 mm and width of 0.7 mm. The data points are fitted to a line through the origin with gradient 0.012 $mS \cdot \mu m^{-1}$.

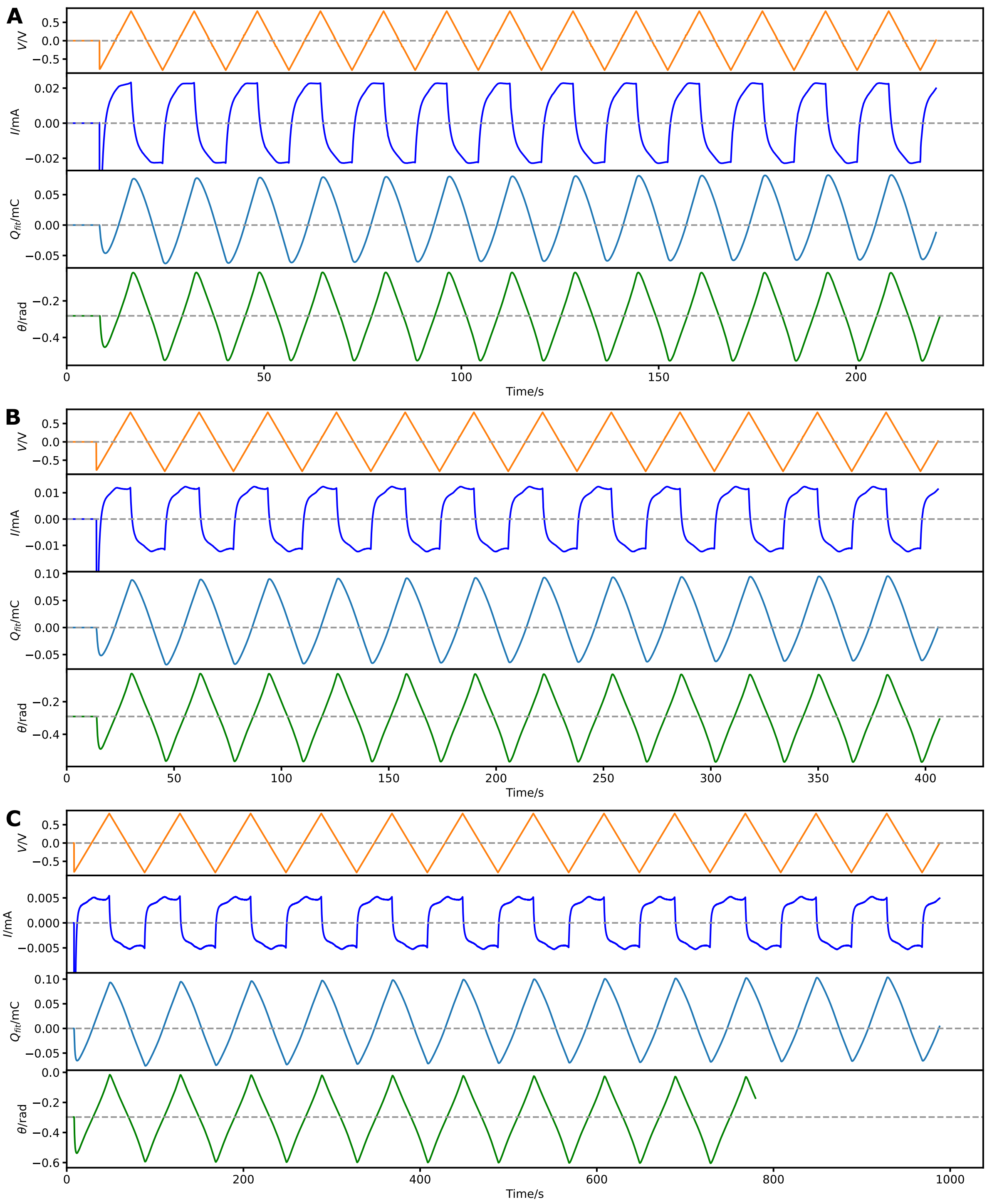

Figure S4. Voltage (V), current (I), charge transfer (Q_{fit}), and bending angle (θ) for CV tests of AJP actuator (Sample II) between ±0.8 V at scan rates of **(A)** 100 mV·s^{-1}, **(B)** 50 mV·s^{-1}, and **(C)** 20 mV·s^{-1}.

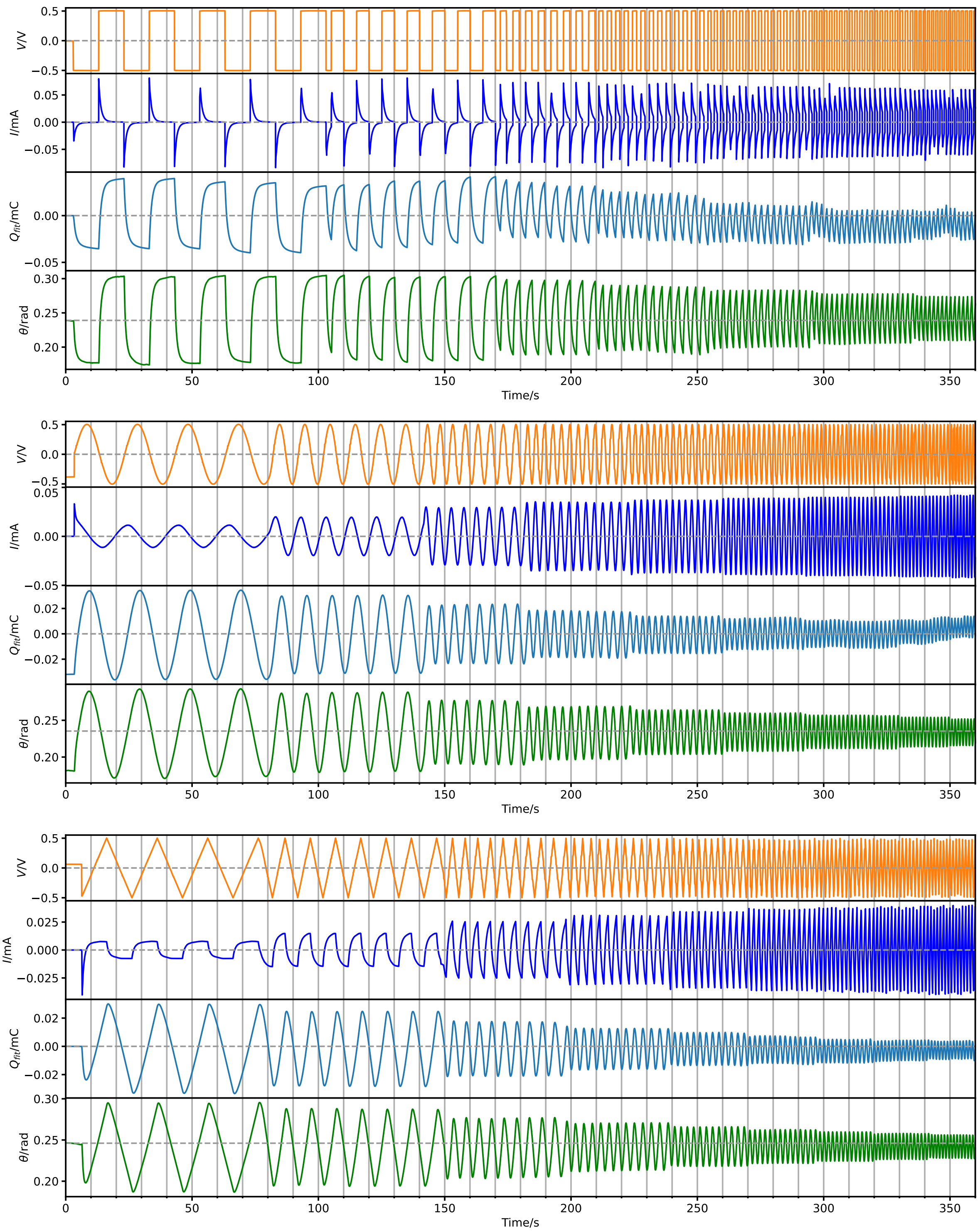

Figure S5. Voltage (V), current (I), charge transfer (Q_{fit}), and bending angle (θ) for frequency sweep tests of AJP trilayer actuator (Sample I) in air from 0.05 Hz to 10 Hz under square, sine, and triangular waves of 0.5 V amplitude.

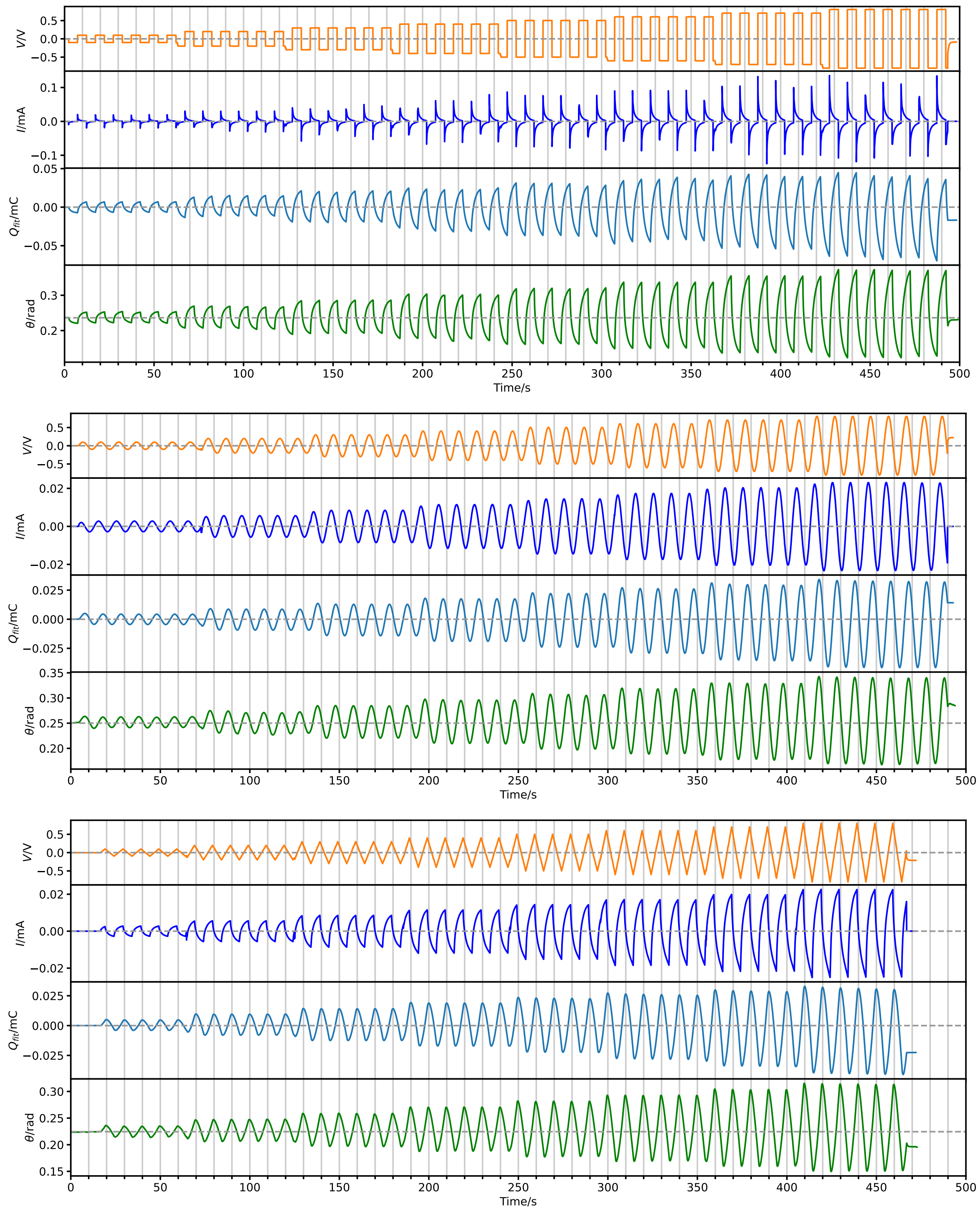

Figure S6. Voltage (V), current (I), charge transfer (Q_{fit}), and bending angle (θ) for voltage sweep tests of AJP trilayer actuator (Sample I) in air from 0.1 V to 0.8 V amplitude under square, sine, and triangular waves at 0.1 Hz.

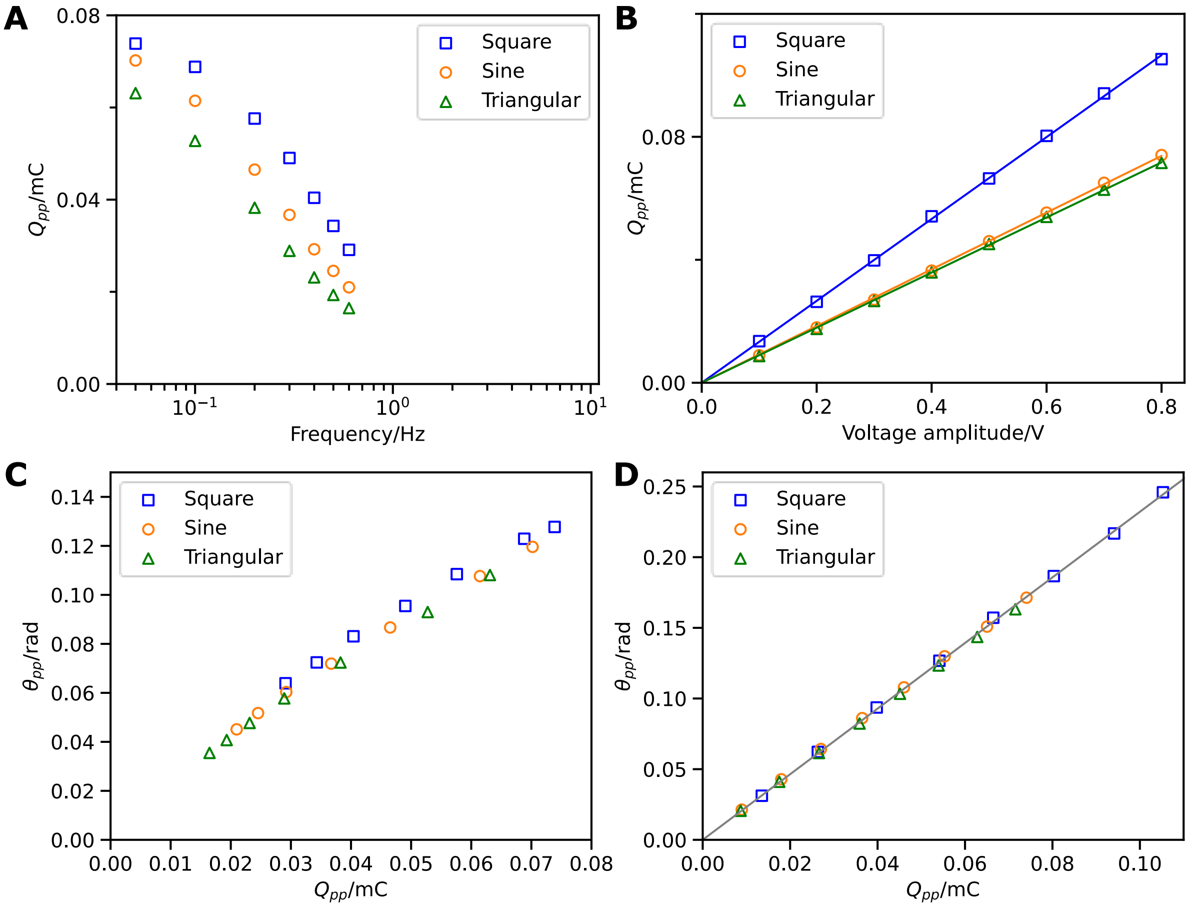

Figure S7. (A) Peak-to-peak charge transfer, Q_{pp}, against frequency for frequency sweep tests at 0.5 V amplitude. **(B)** Q_{pp} against voltage for voltage sweep tests at 0.1 Hz frequency. **(C)** Peak-to-peak deflection, θ_{pp}, against Q_{pp} for the frequency sweep tests. **(D)** θ_{pp} against Q_{pp} for the voltage sweep tests.

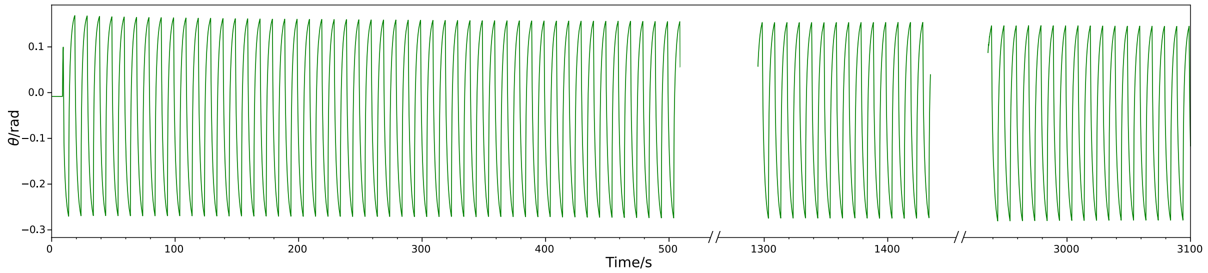

Figure S8. Durability test of AJP actuator (Sample III) under 0.1 Hz, ±0.5 V square wave for 50 minutes.

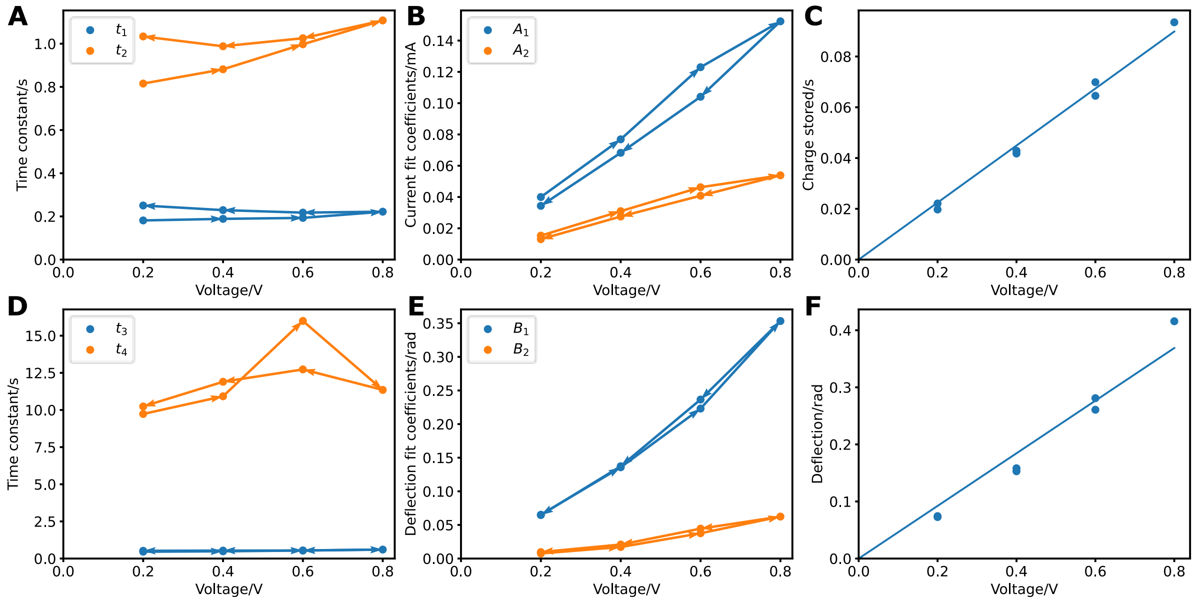

Figure S9. Parameters obtained from fitting each charge-discharge cycle in chronoamperometry test (on Sample II). The arrows in graphs A, B, C, and D indicate the sequence of charging voltages applied. **(A)** Time constants t_1 and t_2 for fitting the current response. **(B)** Coefficients A_1 and A_2 for fitting the current response. **(C)** Maximum charge stored for the charging voltages applied. **(D)** Time constants t_3 and t_4 for fitting the deflection response. **(E)** Coefficients B_1 and B_2 for fitting the deflection response. **(F)** Maximum deflection under the applied charging voltages.

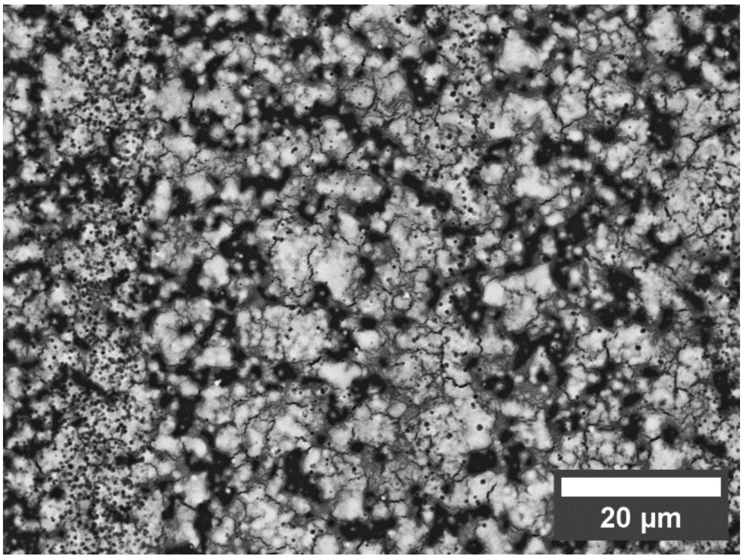

Figure S10. SEM image of AJP Au, showing a microcrack morphology.

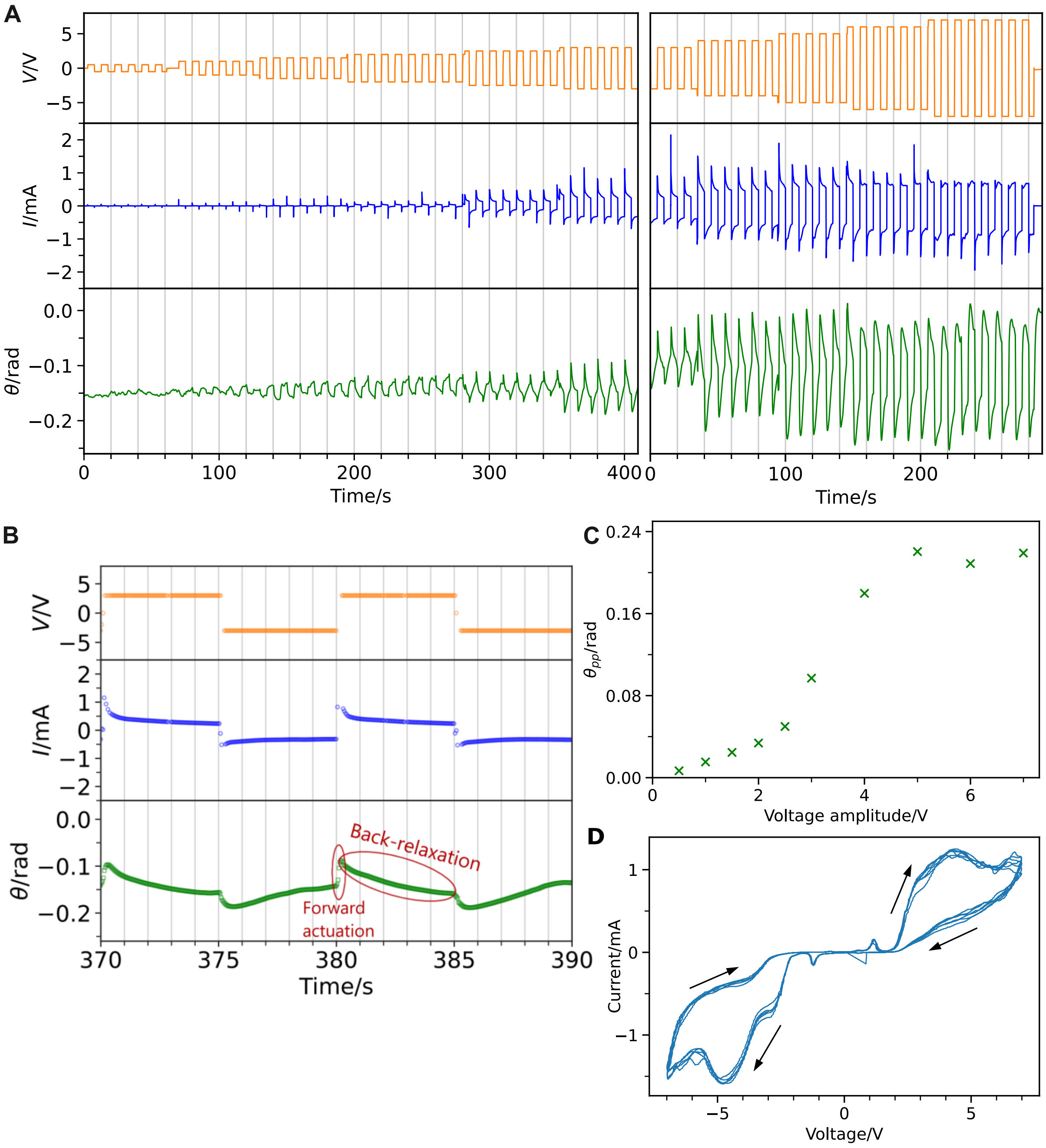

Figure S11. (A) Voltage sweep test of the Au/Nafion/Au actuator in air from 0.5 V to 7 V under square waves at 0.1 Hz. The test is performed in two sessions. **(B)** Zoomed in view showing the back relaxation. After each instance of polarity switch, the motion of the actuator is a fast initial movement towards the anode followed by slow back relaxation towards the cathode. **(C)** Peak-to-peak angular deflection (θ_{pp}) of the Au/Nafion/Au actuator in relation to voltage amplitude of applied to 0.1 Hz square wave. **(D)** Cyclic voltammogram of Au/Nafion/Au actuator measured in air for 5 cycles. The sweep rate is at 60 s per cycle (466.7 mV·s^{-1}).

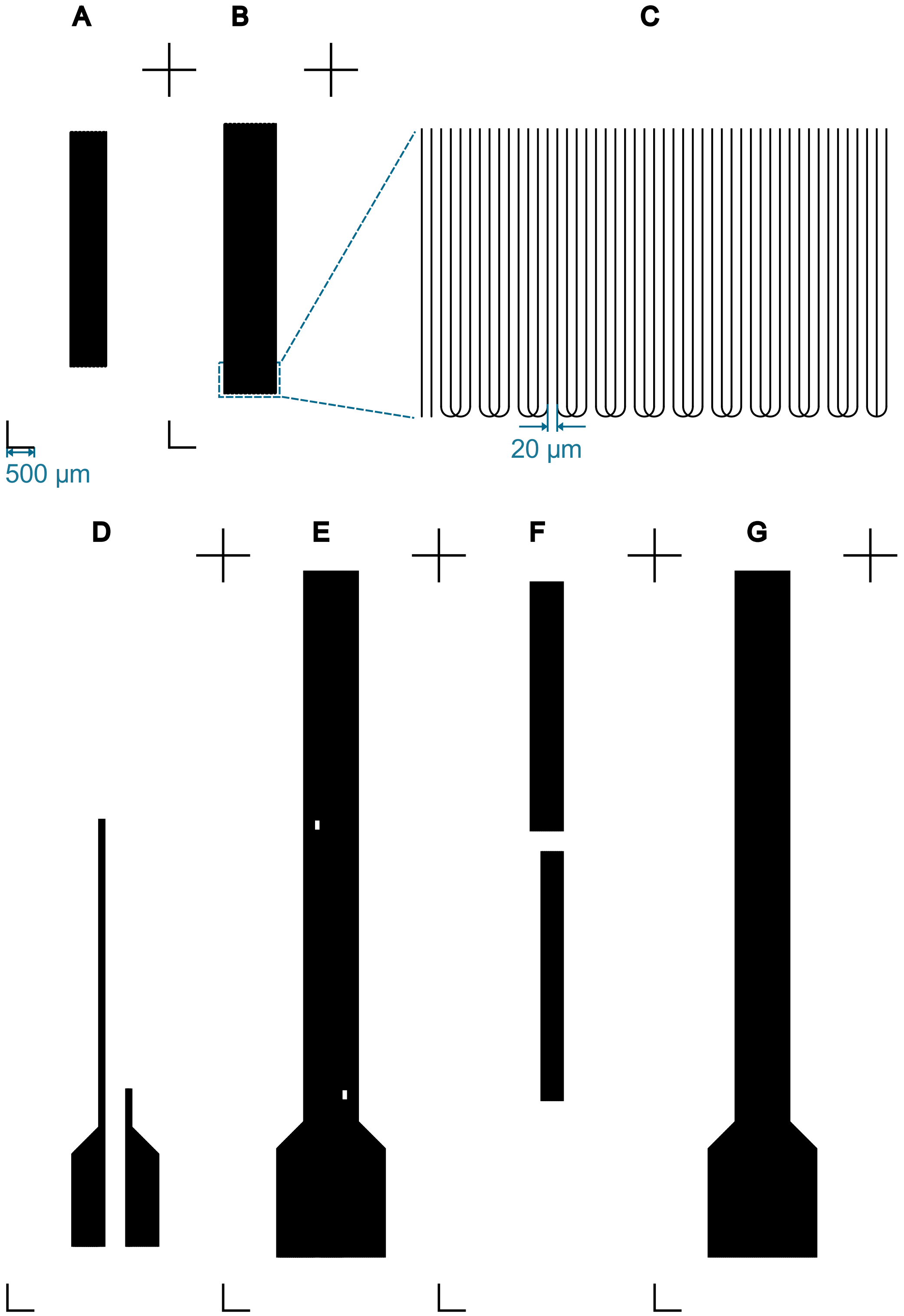

Figure S12. AutoCAD drawings for printing **(A)** the PEDOT:PSS electrode layer and **(B)** the Nafion electrolyte layer of the trilayer actuator. The fiducial markers on the bottom left and top right of each drawing ensure microscale alignment between layers. **(C)** Close-up view of the serpentine lines. In each printing loop, the printer follows two 40 μm-pitch serpentine lines offset by 20 μm. Thus, the raster line separation is effectively 20 μm. **(D-G)** AutoCAD drawings for printing **(D)** Au contact electrodes, **(E)** Nafion encapsulation layers, **(F)** PEDOT:PSS electrodes, and **(G)** the Nafion layer.

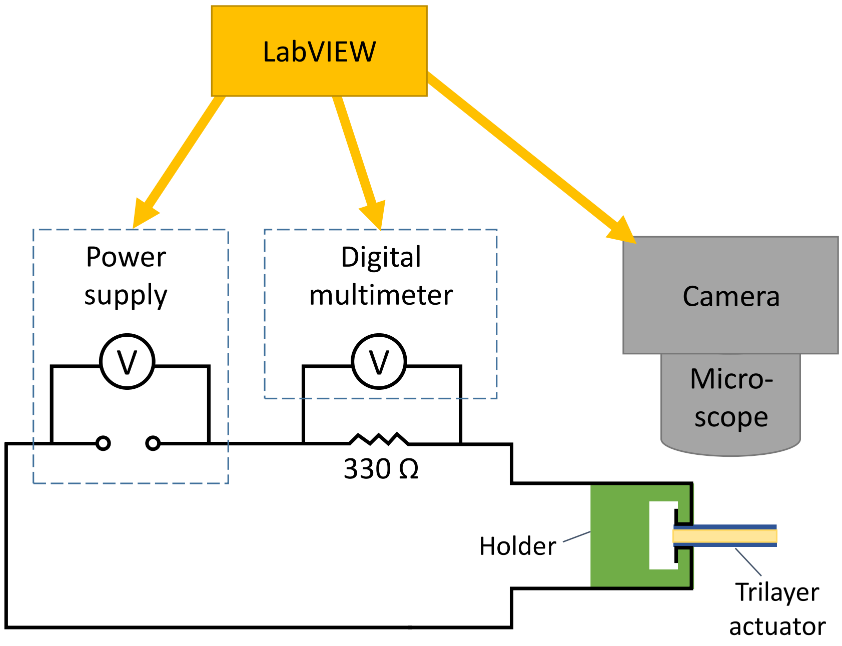

Figure S13. Schematic for actuation testing setup and circuit diagram. A LabVIEW programme controls the bipolar DC power supply, the digital multimeter (DMM), and the camera. The DMM measures the voltage across 330 Ω serial resistor to obtain a reading of current in the circuit. Note that the resistance across the actuator is on the order of ~MΩ. The voltage and current readings and the video are recorded in sync. The power supply can be replaced by a function generator and another DMM to test the actuator under different waveforms.

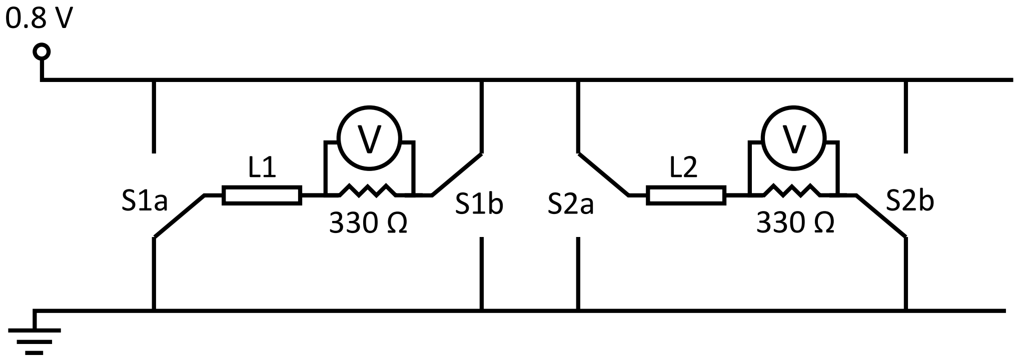

Figure S14. Circuit diagram for controlling a two-segment actuator. The two segments of the actuator are represented by loads L1 and L2. L1 is controlled by a double pole double throw switch, represented in the diagram as two single pole double throw switches S1a and S1b. Similarly, L2 is controlled by switches S2a and S2b. The currents through L1 and L2 are obtained by measuring the voltages across 330 Ω serial resistors using DMMs. The switches can be activated manually. The states of the switches shown in this diagram indicates a scenario where the two segments of the actuator experience opposite current flow, i.e., they are actuated in opposite directions.
